# Individualized cyclic mechanical loading improves callus properties during the remodelling phase of fracture healing in mice as assessed from time-lapsed in vivo imaging

**DOI:** 10.1038/s41598-021-02368-y

**Published:** 2021-11-29

**Authors:** Esther Wehrle, Graeme R. Paul, Duncan C. Tourolle né Betts, Gisela A. Kuhn, Ralph Müller

**Affiliations:** Institute for Biomechanics, ETH Zurich, Leopold-Ruzicka-Weg 4, 8093 Zurich, Switzerland

**Keywords:** Bone, Time-lapse imaging

## Abstract

Fracture healing is regulated by mechanical loading. Understanding the underlying mechanisms during the different healing phases is required for targeted mechanical intervention therapies. Here, the influence of individualized cyclic mechanical loading on the remodelling phase of fracture healing was assessed in a non-critical-sized mouse femur defect model. After bridging of the defect, a loading group (n = 10) received individualized cyclic mechanical loading (8–16 N, 10 Hz, 5 min, 3 × /week) based on computed strain distribution in the mineralized callus using animal-specific real-time micro-finite element analysis with 2D/3D visualizations and strain histograms. Controls (n = 10) received 0 N treatment at the same post-operative time-points. By registration of consecutive scans, structural and dynamic callus morphometric parameters were followed in three callus sub-volumes and the adjacent cortex showing that the remodelling phase of fracture healing is highly responsive to cyclic mechanical loading with changes in dynamic parameters leading to significantly larger formation of mineralized callus and higher degree of mineralization. Loading-mediated maintenance of callus remodelling was associated with distinct effects on Wnt-signalling-associated molecular targets Sclerostin and RANKL in callus sub-regions and the adjacent cortex (n = 1/group). Given these distinct local protein expression patterns induced by cyclic mechanical loading during callus remodelling, the femur defect loading model with individualized load application seems suitable to further understand the local spatio-temporal mechano-molecular regulation of the different fracture healing phases.

## Introduction

Mechanical loading is a key factor for normal progression of the fracture healing process. Delayed fracture repair and non-union formation is a major issue in orthopaedic surgery, with an incidence of 5–10% and a high socioeconomic burden^1,2^ and has been partially attributed to low and inadequate mechanical loading of the defect region^3,4^, affecting all consecutive phases (inflammation, repair, remodelling) of the fracture repair process. The local mechanical conditions in the fracture healing area (for details see tissue differentiation hypothesis by Claes and Heigele^5^) are crucial for the repair process by determining molecular and cellular reactions, the tissue to be formed and the type of ossification. Consequently, modulation of fixation stiffness and (reverse) dynamization have been shown to influence different healing phases^6–10^. We and others have previously shown that externally applied mechanical stimuli, either applied locally to the fracture site via dynamic fixators^11–13^, via tibia loading^14^ or globally via whole body vibration platforms^15,16^, can improve fracture healing. In contrast, inadequate mechanical stimuli resulting in a too low or too high interfragmentary motion were not successful or even impaired the healing process^13,14,17–20^ (for review see^6,21^). In order to understand the underlying mechanisms, refined and well controlled in vivo loading models are needed. Recent studies particularly indicate that profound understanding of the local spatio-temporal mechanical regulation of the fracture healing process seems crucial for the development of safe, targeted and individualized mechanical intervention therapies. Whereas many preclinical fracture healing studies focused on improving the early healing phases (inflammation, repair) with the aim of increasing bone formation and achieving earlier bone union, recent studies also indicate a potential to improve long-bone fracture healing outcome via early modulation of callus remodelling^22,23^ supported by beneficial effects seen with fracture dynamization at the time of cortical bridging^10^. Therefore, the application of controlled external mechanical loading might be suitable to improve and accelerate all phases during fracture healing. However, preclinical fracture healing studies with application of external mechanical loading have so far shown contradictory results^6,21^ and have not been able to completely capture underlying mechanisms due to restrictions in study design: (I) cross-sectional setup with only end-point analyses, (II) effect assessment not specific to healing phases and/or (III) same load for all animals irrespective of individual callus properties and healing progression. We aim to overcome current limitations by combining state-of-the-art time-lapsed in vivo imaging with a novel precisely controlled femur defect loading system in mice. By applying time-lapsed in vivo micro-CT and animal-specific real-time micro-finite element (RTFE) analysis, the developed femur defect loading model allows scaling of loading settings based on strain distribution in the mineralized callus^24^, thereby considering individual differences in healing speed. To follow the healing progression in each animal, a recently developed longitudinal in vivo micro-CT based approach was applied, which was previously shown to capture the different healing phases and to discriminate physiological and impaired healing patterns^25^, without significant radiation-associated effects on callus properties^26^. By registering consecutive scans of the defect region for each animal and implementing a two-threshold approach, data on bone turnover and mineralization kinetics can be obtained, which is particularly important for targeting callus remodelling under different conditions.

The objective of this study was to assess the influence of individualized cyclic mechanical loading on the remodelling phase of fracture healing via longitudinal in vivo micro-CT imaging. For this purpose, we developed a femur defect loading model, that allows application of individualized cyclic mechanical loading based on computed strain distribution in the mineralized callus using animal-specific real-time micro-finite element analysis with 2D/3D visualizations and strain histograms. We hypothesized, that individualized cyclic mechanical loading improves structural and dynamic properties of the mineralized callus during the remodelling phase of fracture healing.

The study showed that the remodelling phase of fracture healing is highly responsive to cyclic mechanical loading leading to significantly accelerated and larger mineralized callus formation and a higher degree of mineralization. Loading-mediated maintenance of callus remodelling was associated with distinct effects on Wnt-signalling-associated molecular targets Sclerostin and RANKL in callus sub-regions and the adjacent cortex of one randomly selected animal per group. The tightly controlled femur defect loading model could in the future widen our knowledge on the local spatio-temporal mechano-molecular regulation of all fracture healing phases.

## Results

**Figure 1:**
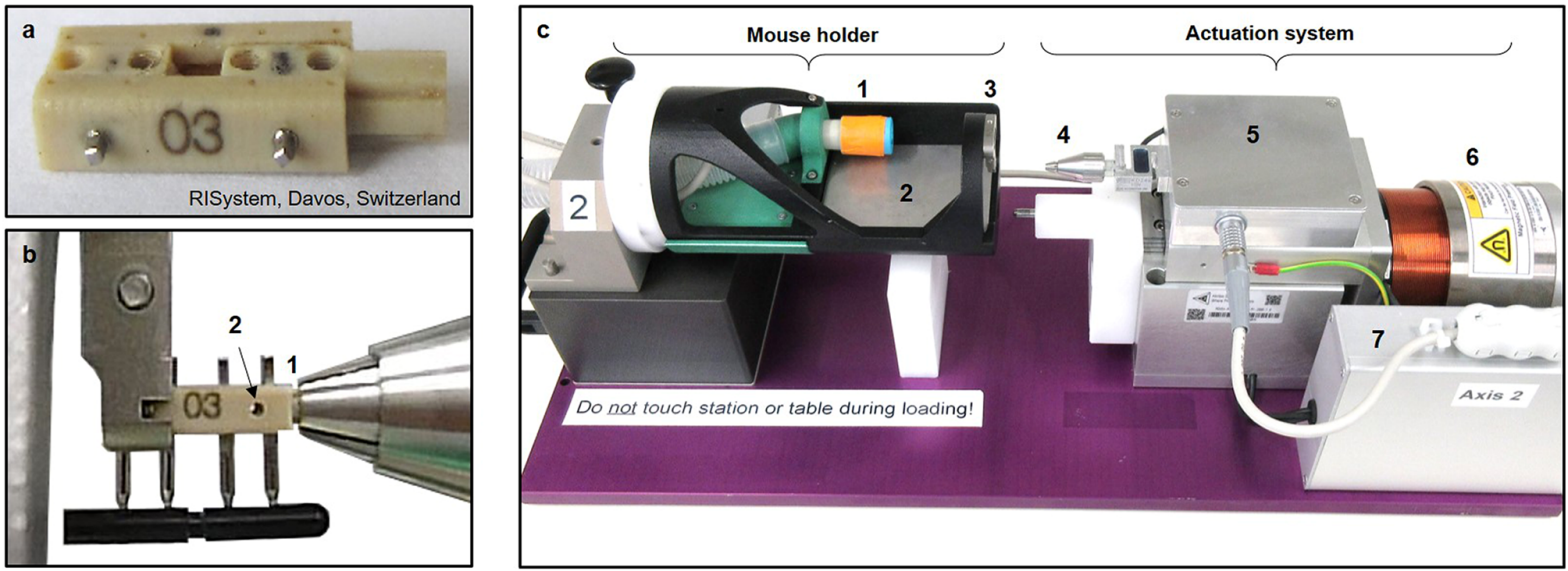
Loading system for femur defects in mice. (**a**) Loading fixator (RISystem, Davos, Switzerland) consisting of four PEEK parts connected via two transverse Titanium pins. (**b**) Insertion of loading fixator into loading device with clamping of loading adapter (1) and removal of transverse pin (2). (**c**) Loading device consisting of mouse holder with anaesthesia inlet (1), heated ground plate (2), clamp for fixator (3) and actuation system with loading chuck (4), load cell electronics box (5), electro-magnetic actuator (6), actuator control electronic box (7). The mouse holder can be used for the loading device and the CT scanner without the need to change the position of the mouse. For detailed description and drawings see Paul et al.^28^.

**Figure 2:**
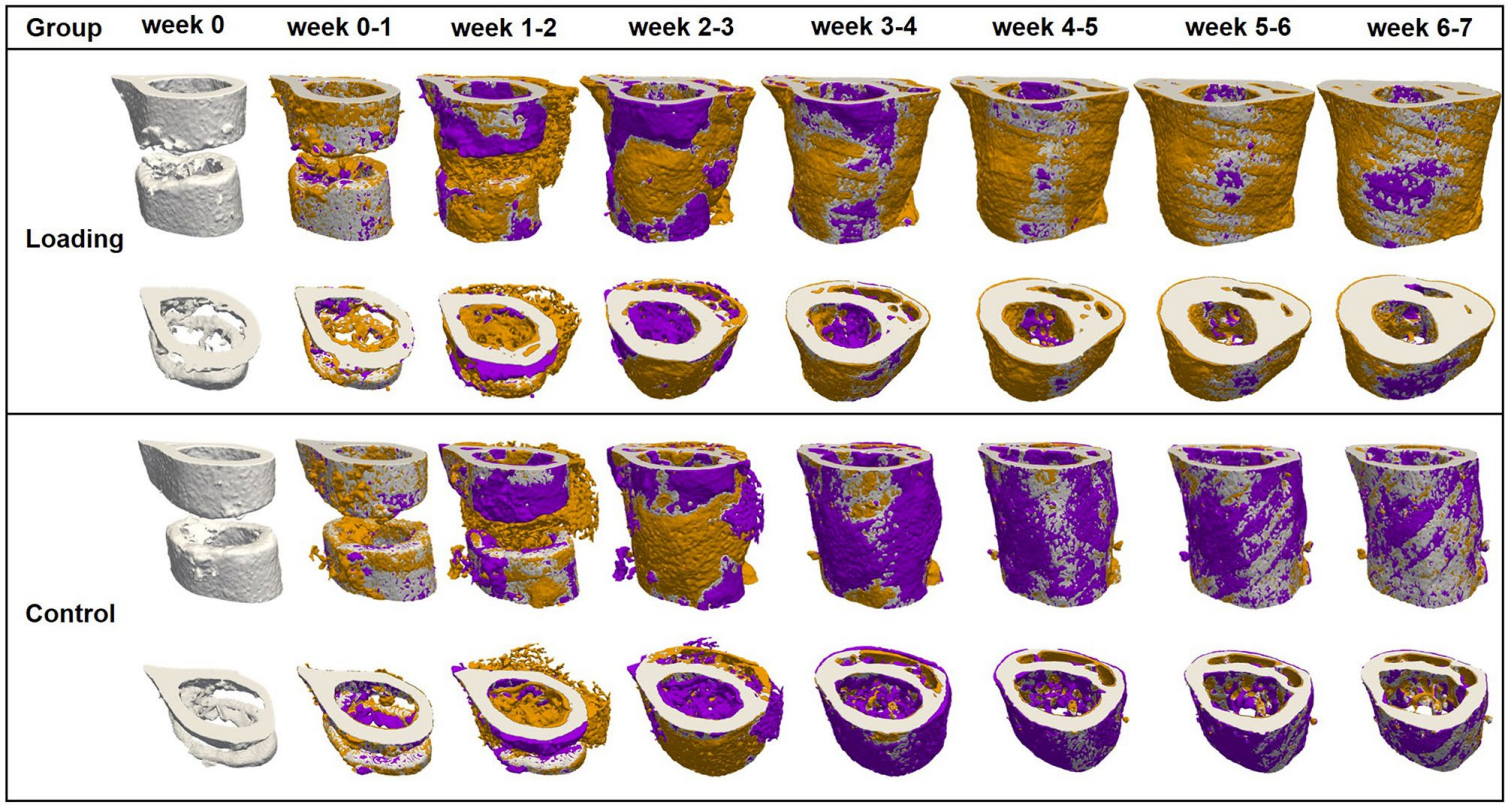
Longitudinal imaging of the defect region via micro-CT. Representative images (full image and cut; threshold: 645 mg HA/cm^3^) of the defect region from animals of the loading group (week 0–7) and the control group (week 0–7). Visualization of bone formation (orange) and resorption (blue) via registration of micro-CT scans from weeks 1–6 to weeks 0–5.

**Figure 3:**
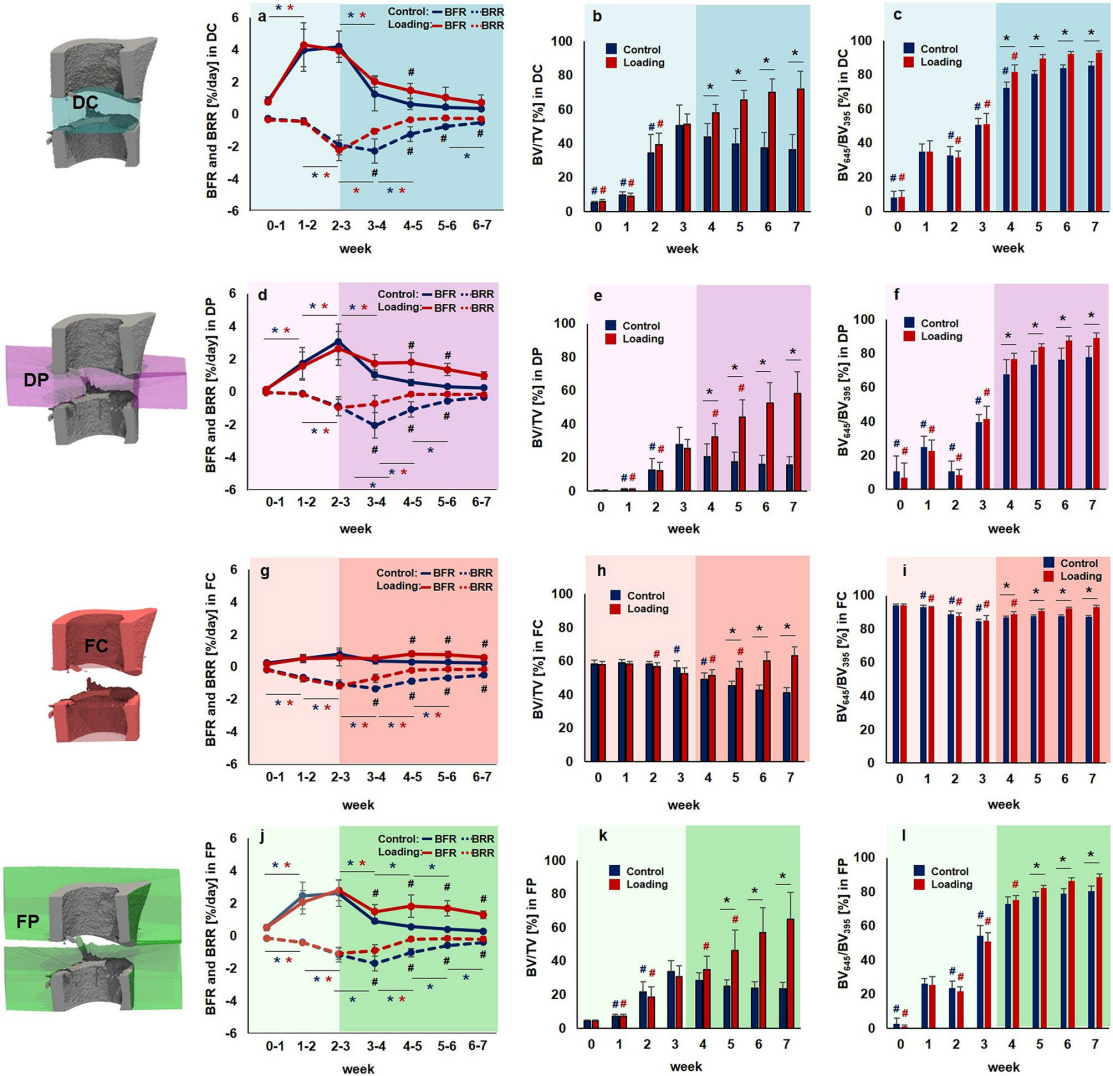
Micro-CT based evaluation of bone parameters in the defect region during the healing period. Four volumes of interest (VOIs) were assessed in the loading group (red) and the control group (blue) from week 0 to week 7 (pre-loading healing period highlighted in lighter colour; loading period highlighted in darker colour): defect center (DC; **a**-**c**), defect periphery (DP; **d**-**f**), cortical fragment center (FC; **g**-**i**), cortical fragment periphery (FP; **j**-**l**). **a**,**d**,**g**,**j**: Bone formation rate (solid line) and bone resorption rate (dashed line) given in percent per day. **b**,**e**,**h**,**k**: Bone volume (BV) normalized to TV (DC for DC and DP, FC for FC + FP). **c**,**f**,**i**,**l**: Degree of bone mineralization given as ratio of bone volume with a density ≥ 645 mg HA/cm^3^ to the total osseous volume (threshold ≥ 395 mg HA/cm^3^). n = 10 per group; *indicates *p* < 0.05 determined by two-way ANOVA with Geisser-Greenhouse correction and Bonferroni correction.

By combining a novel in vivo femur defect loading model (Fig. 1) with a recently established time-lapsed in vivo micro-CT based monitoring approach^26,27^, the influence of individualized cyclic mechanical loading on properties of the mineralized callus was assessed during the remodelling phase of fracture healing. A loading group (n = 10) received individualized cyclic mechanical loading (8–16 N, 10 Hz, 5 min, 3 × /week) based on computed strain distribution in the mineralized callus using animal-specific RTFE, whereas controls (n = 10) received 0 N for 5 min at the same post-operative time-points. By registration of consecutive scans, structural and dynamic parameters in the mineralized callus were followed in three callus sub-volumes (defect centre: DC, defect periphery: DP, cortical fragment periphery: FP, and the adjacent cortical fragments: FC over time (Figs. 2, 3). For details on methods see Tourolle né Betts et al.^27^; for detailed study design see Supplementary Table S1.

### General physical observation

All mice recovered rapidly from surgery and there were no dropouts in both groups (loading, control) during the complete experimental period. The body weight of the animals did not significantly change over time (see Supplementary Fig. S1). Similar to observations from a previous femur defect study^25^, nest building started on the day of surgery and all animals were found in groups in the nest in the morning of post-operative day 1. In both groups, social interaction between mice and nesting behaviour during the pre-loading healing period (week 0–3) and the loading period (week 3–7) did not differ from pre-surgical observations.

### Volumes of interest (VOI) for evaluation by time-lapsed in vivo micro-CT

In order to exclude bias in the further micro-CT analyses, we compared the size of the defects and different VOIs (depicted in Fig. 3; for details on VOI generation see Tourolle né Betts et al.^27^). The defect sizes were 0.63 ± 0.07 mm for the control and 0.60 ± 0.07 mm for the loading group (n = 10/group). The VOIs encompassed the following volume for the control (n = 10) and the loading group (n = 10): 1.14 ± 0.13 mm^3^ versus 1.04 ± 0.16 mm^3^ for the defect centre (DC), 6.68 ± 0.77 mm^3^ versus 6.35 ± 0.82 mm^3^ for the defect periphery (DP), 2.28 ± 0.17 mm^3^ versus 2.31 ± 0.16 mm^3^ for the cortical fragments (FC), 15.44 ± 0.70 mm^3^ versus 15.84 ± 0.99 mm^3^ for the fragment periphery (FP). No significant differences in volume were detected in any of the VOIs between groups.

### Pre-loading healing patterns monitored by time-lapsed in vivo micro-CT

In the pre-loading healing period (week 0 to week 3) similar and physiological healing patterns were observed for the control group (n = 10) and the loading group (n = 10) in all defect VOIs (DC, DP and FP) and the adjacent cortex (FC; Figs. 2, 3). Registration of consecutive in vivo micro-CT scans allowed to capture distinct characteristics of the mineralized callus indicative of the different healing phases (inflammation, repair, remodelling; significant weekly differences in bone parameters indicated in Fig. 3). From week 0–1 to week 1–2 in both groups a significant 4 × to 14 × increase in bone formation was detected in the three callus VOIs DC (control group: *p* = 0.0007; loading group: *p* = 0.0006; Fig. 3a), DP (control group: *p* < 0.0001; loading group: *p* < 0.0001; Fig. 3d) and FP (control group: *p* = 0.0012; loading group: *p* = 0.0006; Fig. 3j), indicating progression from the inflammation to the reparative phase. This led to a significant gain in bone volume (BV/TV) by week 2 in the callus VOIs DC (control group: *p* < 0.0001; loading group: *p* < 0.0001; Fig. 3b), DP (control group: *p* < 0.0001; loading group: *p* < 0.0001; Fig. 3e) and FP (control group: *p* < 0.0001; loading group: *p* < 0.0001; Fig. 3k). From week 1–2 to week 2–3 a significant 3 × to 9 × increase in resorptive activities was seen in the callus VOIs DC (control group: *p* = 0.0023; loading group: *p* < 0.0001; Fig. 3a), DP (control group: *p* = 0.0499; loading group: *p* = 0.0162; Fig. 3d) and FP (control group: *p* = 0.0326; loading group: *p* = 0.0070; Fig. 3j), indicating the progression from the repair to the remodelling phase. From week 2 to week 3, the highly mineralized bone fraction in the callus VOIs significantly increased (*p* < 0.0001 in DC, DP, FP) in both groups, indicating callus maturation (Fig. 3c,f,l).

From week 0 to week 3, in both groups, strong resorptive activities (Figs. 2, 3g) were seen in the adjacent cortical fragments (FC). BRR showed significant weekly increases whereas no significant weekly changes in bone formation were observed in this VOI. The strong resorptive activities let to decreased bone volume by week 3 reaching statistical significance in the loading group (*p* < 0.0001). Furthermore, the fraction of highly mineralized bone significantly decreased from week 1 to week 3 in both groups (*p* < 0.0001), indicating cortical reorganisation.

### Individualized cyclic mechanical loading improves properties of the mineralized callus during the remodelling phase of fracture healing

Individualized cyclic mechanical loading (8–16 N, 10 Hz, 300 cycles, 3 × /week) started after bridging of the femur defect (week 3; Table 1) at the beginning of the remodelling phase (loading group, n = 10). RTFE allowed determination of initial loads (8–12 N) based on individual callus properties, weekly load-scaling (8-16 N) and matching of strain distribution in the mineralized callus for the 4-week loading period. Control animals received 0 N loading at the same post-operative time-points. Already after 1 week, the first loading-associated effects on properties of the mineralized callus and the adjacent cortical bone were seen: Specifically, loading reduced BRR in all VOIs in week 3–4 compared to pre-loading values in week 2–3 (Fig. 3a,d,g,j) reaching statistical significance in DC (*p* < 0.0001). In contrast, in the control group, BRR further increased in all VOIs during the same period with statistical significance seen in the peripheral VOIs (*p* = 0.0066 for DP, *p* = 0.0126 for FP). From week 3–4 to week 6–7, the applied individualized mechanical loading was also able to maintain bone formation at significantly higher levels compared to controls, while bone resorption was significantly reduced during the same period. This led to a linear increase in osseous callus volume in loaded animals from week 3 to week 7 in all callus VOIs (DC: + 41%, DP: + 130%, FP: + 111%) and the adjacent cortex (FC: + 20%), whereas it significantly declined in controls (DC: − 28%, DP:  − 44%, FP:  − 27%, FC:  − 30%). In week 6–7, no significant differences in bone turnover were seen anymore indicating load adaptation. After 7 weeks, loaded animals had significantly more bone in the defect (2 × for DC, *p* < 0.0001; 4 × for DP, *p* < 0.0001; 3 × for FP, *p* < 0.0001) with a significantly larger fraction of highly mineralized bone compared to controls (+ 8% for DC, *p* = 0.0006; + 15% for DP, *p* < 0.0015; + 10% for FP, *p* < 0.0001). Loading was associated with maintenance of the endosteal callus throughout the assessed healing period, while the medullary cavity was largely restored in controls (see Supplementary Fig. S3 for callus cross-sections of all animals). Three-point bending also showed a 46% higher relative bending stiffness (healed femur vs. contralateral femur) in the loaded animals (107%) compared to controls (73%; n = 2/group; Supplementary Fig. S2). In addition, according to the standard clinical evaluation of X-rays, the number of bridged cortices per callus was evaluated in two perpendicular planes and animals with ≥ 3 bridged cortices were categorized as healed. From week 4 to week 7 all defects in both groups were categorized as healed (Table 1), indicating that the applied forces during cyclic mechanical loading did not lead to re-fracture of the mineralized callus.

**Table 1:** Fracture healing outcome assessed by cortical bridging (healed fracture ≥ 3 bridged cortices, threshold 395 mg HA/cm^3^).

		Week							
	Group (n = 10)	0	1	2	3	4	5	6	7
Healed fractures per group	^Control^⁄_Loading_	^0^⁄_0_	^0^⁄_0_	^5^⁄_6_	^9^⁄_10_	^10^⁄_10_	^10^⁄_10_	^10^⁄_10_	^10^⁄_10_

Loading also affected the bone turnover in the adjacent cortex (FC). Already after 1 week, loaded animals showed significantly lower cortical bone resorption compared to controls (Fig. 3j). Whereas cortical volume significantly declined (*p* < 0.0001) in control animals from week 3 to week 7 by 27%, it significantly increased (*p* < 0.0001) by 20% in the loaded animals during the same time period (Fig. 3k). From week 4 to week 7 loading was also associated with significantly higher cortical mineralization (+ 4% in week 4 to + 54% in week 7) compared to controls (week 4: *p* = 0.0459, week 5–week 7: *p* < 0.0001; Fig. 3l).

In week 7, the FC VOI comprised 46% and 34% of the osseous tissue in the total VOI (TOT) for the control and loading group, respectively. In the DC VOI, 20% (control group) and 17% (loading group) of the total bone volume were seen. In the two peripheral VOIs, 9% (control group) and 14% (loading group) of the total osseous tissue were seen in the DP and 26% (control group) and 35% (loading group) in the FP VOI. Loading was associated with a shift in the bone distribution from the cortical fragments to the peripheral VOIs.

### Histology

**Figure 4:**
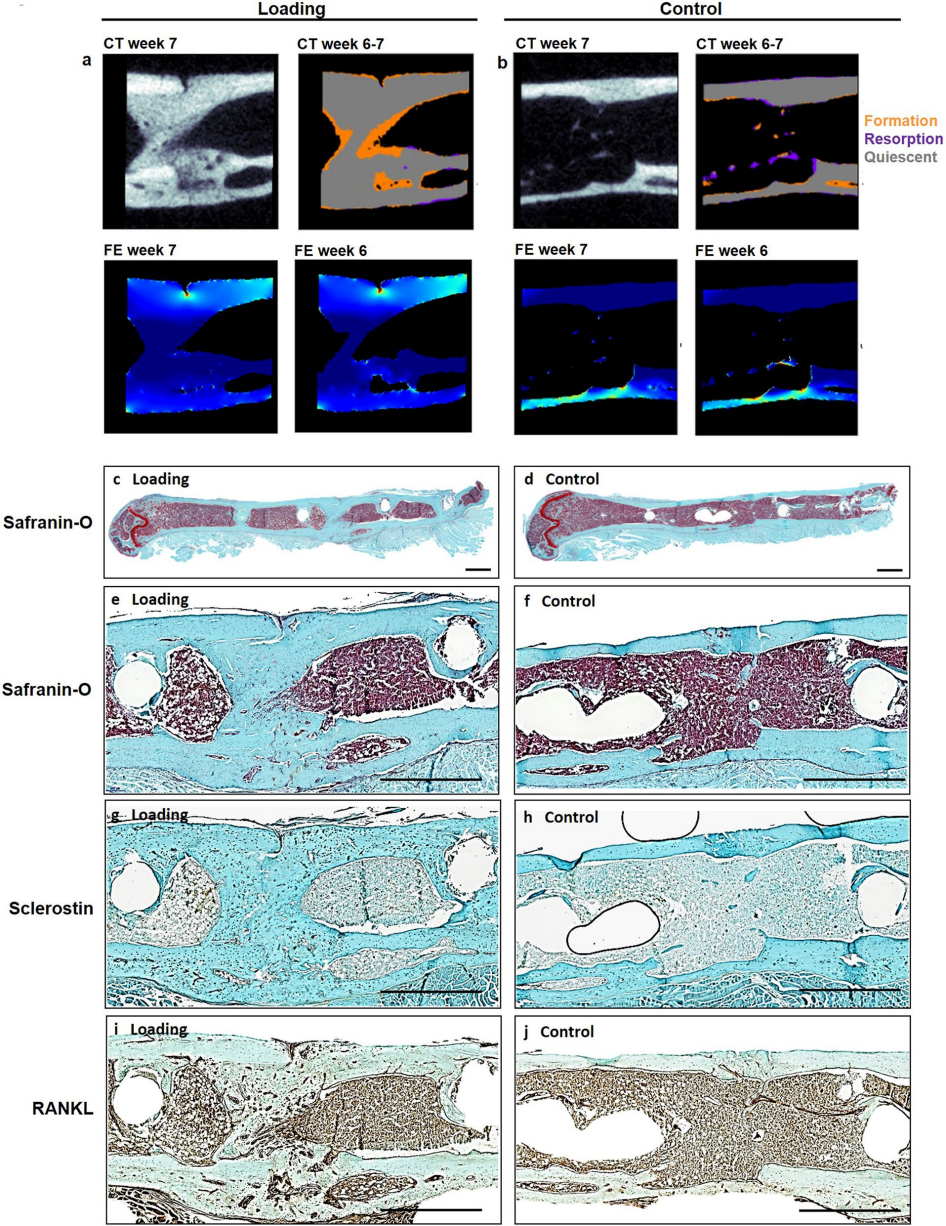
Micro-CT and histological analyses of consecutive callus sections. (**a**, **b**): Micro-CT images of fracture callus sections corresponding to sections stained with Safranin-O for one animal from the loading (**a**) and the control group (**b**): CT image (threshold: 395 mg HA/cm^3^), visualization of regions of bone formation and resorption via registration of CT image from weeks 6 and 7, visualization of strains on CT section via micro-FE analysis for week 7 and week 6. (**c**-**j**). Consecutive longitudinal sections of fractured femora 7 weeks after defect surgery of one animal from the loading group and the control group stained for Safranin-O (**c**-**f**), Sclerostin (**g**, **h**), RANKL (**i**, **j**). Scale bar = 100 µm (**c**, **d**), scale bar = 500 µm (**e**-**j**).

In order to also visualize the tissue composition of the callus, we performed end point histological stainings of serial sections from one randomly selected animal of each group. Histology supported our micro-CT findings with complete cortical bridging seen in the animal from the loading and from the control group (Fig. 4b-i). The loaded animal showed a larger callus with some cartilage fractions suggesting ongoing endochondral ossification processes, whereas in the control animal the callus was largely remodelled with restoration of the medullary cavity indicating proceeding towards the end of healing (Fig. 4b-e) further supporting the observations from micro-CT cross-sections (n = 10/group) shown in Supplementary Fig. S3.

**Figure 5:**
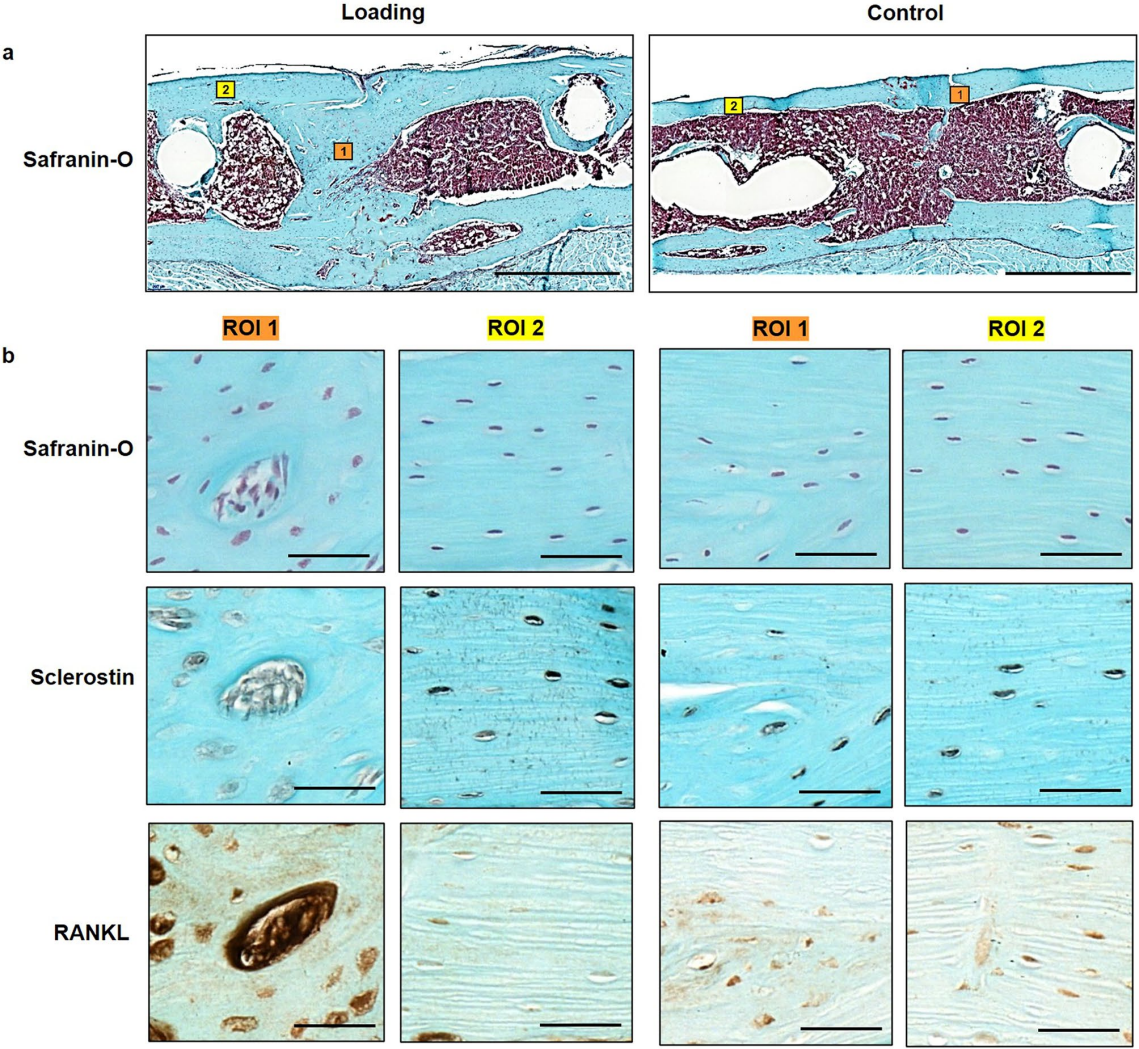
Visualization of local cellular distribution patterns and protein expression in the fracture callus and adjacent cortex. (**a**) Longitudinal Safranin-O stained sections of fractured femora 7 weeks after defect surgery of one animal from the loading group and one animal from the control group. Boxes indicate regions depicted in (**b**) (1: callus; 2: cortex); Scale bar = 100 µm. (**b**) Magnification of callus region (left) and cortical region (right) stained for Safranin-O, Sclerostin and RANKL; Scale bar = 50 µm.

Furthermore, differences in the shape of the cell nuclei were observed between the osteocytes in the fracture callus (round shape) of the loaded animal in comparison to the cell nuclei seen in the cortical bone of the same animal (ellipsoidal shape) and the cell morphology seen in the control animals (Fig. 5a + b), suggesting an association between the local mechanical environment and cellular morphology. In regions with lower strains visualized by micro-FE (endosteal fracture callus; Figs. 4a, 5b), rounder cell nuclei were observed, compared to ellipsoidal nuclei shape in higher strained regions (cortex; Figs. 4a, 5b). To capture potential underlying mechano-molecular targets, we performed immunohistochemistry of Sclerostin (inhibitor of the mechano-responsive and osteoanabolic Wnt signalling pathway) and RANKL (negatively-regulated target gene of Wnt signalling) which have both previously been associated with the mechanical regulation of bone adaptation and healing^16,29–33^. Less abundant Sclerostin staining was visible in the fracture callus compared to the cortical bone of the loaded animal (Fig. 4 f and 5b) with no region-specific differences in staining patterns being seen in the control animal (Figs. 4g, 5b), suggesting that Wnt-signalling contributes to the loading-mediated osteoanabolic effects on fracture healing seen in this study. At the same time RANKL, which is down-regulated by Wnt-signalling, was more abundantly and stronger expressed in the callus of the loaded animal compared to the control animal (Fig. 4h + i and 5b). In line with previous studies, strong RANKL expression was seen in lowly strained callus regions as visualized via micro-FE analysis (Figs. 4a, 5b), indicating ongoing bone remodelling.

## Discussion

In this study, the effect of individualized cyclic mechanical loading on the remodelling phase of fracture healing was assessed in a novel femur defect loading model in mice using a recently developed in vivo micro-CT based monitoring approach. Given the strong mechano-responsiveness of bone cells, several studies have applied mechanical loading (e.g. globally via vibration platforms, using tibia loading or locally via external fixators) to improve fracture healing showing contradictory results^6,11–21^. Whereas some studies saw a loading-mediated improvement of fracture healing^6,11–16,21^, others were not successful or even reported an impairment of the fracture healing process^13,14,17–20^. One limitation of these studies was the application of the same load to all animals thereby not taking into account individual differences in healing progression associated with age and estrogen status of the animals which might have resulted in insufficient load application to some animals. In order to allow for individualized mechanical loading we now developed a precisely controlled in vivo loading model based on external fixation of femur defects in mice. This approach also minimizes the risk of re-fracturing lowly mineralized bone after initial bridging of the defect. By combining state-of-the-art time-lapsed in vivo imaging with individualized mechanical loading protocols adapted to animal-specific properties of the mineralized callus, this allows to precisely characterize spatio-temporal effects of mechanical loading on the fracture callus.

Via weekly in vivo micro-CT imaging, we saw, that cyclic mechanical loading significantly augmented the formation of mineralized callus early after initial cortical bridging and subsequently, linearly increased the osseous callus volume during the remodelling phase of fracture healing. By the study end, loaded animals had significantly more bone in all callus VOIs (2 × for DC, *p* < 0.0001; 4 × for DP, *p* < 0.0001; 3 × for FP, *p* < 0.0001) with a significantly larger fraction of highly mineralized bone compared to controls (+ 8% for DC, *p* = 0.0006; + 15% for DP, *p* < 0.0015; + 10% for FP, *p* < 0.0001). This is supported by recent study from Liu et al.^34^ describing additional loading-mediated bone formation during the remodelling phase in a uni-cortical tibia defect model in mice. However, they failed to show statistical significance between the loading and the control group in histomorphometry and micro-CT analyses, which they attributed to the high variability within groups. As the study applied the same load to all animals irrespective of healing progression, differences in the induced strains might have caused the high variability within groups. We addressed this by using our recently developed RTFE based loading approach^24^, which takes into account the individual strain distribution within the mineralized callus for determining individual loading settings at weekly post-operative intervals. In a recent study by Malhotra et al.^35^ individualized cyclic mechanical loading (week 0–week 4, 3 × /week) was shown to significantly increase bone volume fraction in a partial vertebral defect model compared to controls. So far, no study using a complete defect model assessed the effect of local cyclic mechanical loading (e.g. via external fixators, tibia/ulna-loading) when exclusively applied during the remodelling phase of the healing process. An earlier tibia defect loading study (defect size: 3 mm) in sheep by Goodship et al.^36^ applied cyclic mechanical loading (25 µm interfragmentary movement, 30 Hz) throughout the healing period (week 0—week 10, 5 × per week) and reported a significantly larger callus with higher mineralization in loaded animals compared to controls. However, as the loading application was not restricted to specific post-operative time frames, it was not possible to evaluate whether loading during a specific fracture healing period was sufficient for the observed improvement in fracture healing or if cyclic mechanical loading needs to be applied throughout the healing process to achieve such an effect. We now showed, that the remodelling phase is highly responsive to cyclic mechanical loading applied after initial cortical bridging of the defect, with pronounced effects on callus size and mineralization. This is in line with studies showing that increasing the interfragmentary movement via dynamization at the time of cortical bridging can improve healing outcome^10^. Via limiting the loading application to the earlier healing period (day 7–day 19), Gupta et al.^11^ aimed at assessing (histomorphometry and radiography on day 42) whether there is a sustained effect of cyclic mechanical loading (2%, 10% and 30% strain, 0.5 Hz, 500 cycles) on fracture healing outcome. They found a larger bone fraction in the callus and higher cortical bridging scores in the loaded compared to the control animals, indicating, that loading effects persisted after a 4-week non-loading period. In contrast to these studies, Schwarz et al.^17^ did not observe any loading-mediated improvement on fracture healing in a critically-sized femur defect model in rats (defect size: 5 mm). However, in this study loading (6 cycles of constant loading at 500 µm IFM and 40 s rest phase), was only applied once per week, which indicates a critical role of the type of loading (constant vs. cyclic) and the frequency of loading sessions per week. Whether cyclic mechanical loading in contrast to constant mechanical loading is able to prevent non-union formation in critically-sized defects needs to be assessed in future studies.

We were now able to also include dynamic parameters such as bone formation and resorption in the monitoring of loading-mediated effects on fracture healing via registration of longitudinal in vivo micro-CT scans (for details see Tourolle né Betts et al.^27^). Compared to histology-based determination of the bone formation rate via in vivo application of fluorescent dyes (e.g. calcein, alizarin), our micro-CT-based monitoring approach with registration of consecutive scans also allows for assessment of the bone resorption rate in individual animals, which was shown to be particularly suited for the characterization of the remodelling phase during the fracture healing process^25,26^. The most striking loading-mediated effect in the current study was the prompt and significant reduction in bone resorption rate (week 3–4 vs. week 2–3), which was seen in the central and periosteal VOIs (DC, FP) as early as 1 week after loading (Fig. 3a,j). Compared to control animals, in which bone resorption still increased during this early remodelling period (week 3–4), loaded animals showed significantly lower bone resorption rates in all callus VOIs (DC, DP, FP) in this first week of loading, indicating a strong mechano-responsiveness of the fracture callus during this healing period. Most of the previous studies either assessed the loading effects only after a longer loading period of minimum 2 weeks^17,37^ or they did not see significant loading-mediated effects on the fracture callus after shorter treatment periods^11,16,19^. So far only one study by Leung et al.^38^ was able to detect a loading-mediated improvement of fracture healing (larger callus diameter) after a short loading period of 1 week. We were now able to strengthen these cross-sectional structural callus findings with our longitudinal micro-CT data obtained for single animals. In respect to the underlying mechanisms, we showed for the first time, that not only bone formation but also bone resorption can be significantly modulated via cyclic mechanical loading during fracture healing, indicating a potential for mechanical intervention therapies to improve impaired fracture healing conditions associated with imbalanced bone remodelling. Furthermore, implementation of our two-threshold approach showed cyclic mechanical loading to be also effective in significantly advancing callus mineralization compared to control animals. Via combining our novel loading model with time-lapsed in vivo imaging and a two-threshold approach, we were able to characterize the spatio-temporal effects of loading during the remodelling phase of fracture healing. Furthermore, the applied micro-FE-based loading approach also allowed for spatio-temporal visualization of strains induced in the fracture callus (Fig. 4a + b). In order to also visualize the tissue composition of the callus, we performed end point histological stainings of serial sections from one animal of each group. Histology supported our micro-CT findings with complete cortical bridging seen in both groups. However, the loaded animal showed a larger callus with some cartilage fractions suggesting ongoing endochondral ossification processes, whereas in the control animal the callus was largely remodelled with restoration of the medullary cavity indicating proceeding towards the end of healing. This was supported by micro-CT showing maintenance of endosteal callus in loaded and restoration of the medullary cavity in control animals (Supplementary Fig. S3). Furthermore, differences in the shape of the cell nuclei were seen between the osteocytes in the fracture callus (round shape) of the loaded animal in comparison to the cell nuclei seen in the cortical bone of the same animal (ellipsoidal shape) and the cell morphology seen in the control animals. This is particularly interesting, as previous in vitro studies found, that round cells are more sensitive to mechanical loading compared to flat and elongated cells^39–41^, suggesting that repeated cyclic mechanical loading might be able to induce round cellular morphology of (early) osteocytes, thereby maintaining the remodelling phase of fracture healing eventually leading to higher mineralization of the newly formed tissue compared to controls. In the in vivo setting osteocytes reside in lacunae within the lacunocanalicular network (LCN). A recent study by Casanova et al.^42^ longitudinally assessed the development and evolution of the LCN during fracture healing indicating a progressive increase in the complexity of the LCN, which they attributed to factors expressed by osteocytes such as matrix metalloproteinases, Sclerostin and RANKL. To capture potential underlying molecular targets of the spatio-temporal loading effects seen in the current study, we performed immunohistochemistry of Sclerostin (inhibitor of the mechano-responsive and osteoanabolic Wnt signalling pathway) and RANKL (negatively-regulated target gene of Wnt signalling) which have both previously been associated with the mechanical regulation of bone adaptation and healing^16,29–33^. Whereas visual inspection showed less abundant Sclerostin staining in the fracture callus compared to the cortical bone of the loaded animal (Figs. 4f, 5b), no region-specific differences in staining patterns were seen in the control animal (Figs. 4g, 5b). This might indicate that Wnt-signalling contributes to the loading-mediated osteoanabolic effects on fracture healing seen in this study. However, at the same time we saw more and stronger RANKL staining in the callus of the loaded animal compared to the control animal (Fig. 4h + i). This finding precludes that the loading-mediated increases in RANKL expression seen in this study were solely mediated via Wnt-signalling but rather an interplay with further signalling pathways must have triggered this strong response in RANKL production. Via micro-FE analysis, we saw high RANKL expression in the lowly strained endosteal fracture callus, indicating ongoing bone remodelling. In order to shed light into the complex interplay between a multitude of mechano-responsive molecules and signalling pathway, novel advents in RNAsequencing including spatial transcriptomic approaches might, in future studies, open interesting possibilities to understand the spatio-temporal mechanomics of fracture healing.

Limitations of this study include the low sample number for histology (n = 1/group), and the use of 3-point-bending instead of torsional testing to failure of femora due to restrictions in equipment and sample preservation requirements. Loaded animals showed maintenance of a large endosteal callus, indicative of prolonged remodelling potentially also affecting other skeletal sites due to high calcium consumption. Future studies could focus on shorter loading intervals at the beginning of cortical bridging, in order to accelerate callus formation as seen in this study but at the same time avoiding prolonged maintenance of endosteal callus. The loading-associated higher degree of callus mineralization seen in this study should in the future be further followed up via assessing the remodelling of woven to lamellar bone (e.g. via FTIR). Furthermore, future studies should assess, if the loading-mediated augmentation of callus formation and mineralization is associated with earlier and increased fracture stability potentially allowing for earlier fixator removal compared to non-loaded animals. Via incorporating material properties for non-mineralized tissues into the RTFE pipeline, the effect of individualized cyclic mechanical loading could also be assessed in earlier healing periods.

In summary, using our recently developed femur defect loading model in combination with time-lapsed in vivo micro-CT and a two-threshold approach, we showed that the remodelling phase of fracture healing is highly responsive to cyclic mechanical loading with changes in dynamic callus parameters leading to larger callus formation and mineralization. Loading-mediated maintenance of callus remodelling was associated with distinct effects on the Wnt-signalling-associated molecular targets Sclerostin and RANKL in callus sub-regions and the adjacent cortex as assessed via immunohistochemistry in one randomly selected animal per group. Given these distinct local protein expression patterns induced by external cyclic mechanical loading, our tightly-controlled femur defect loading model with individualized load application could be used in future studies to precisely understand the local spatio-temporal mechano-molecular regulation of the different fracture healing phases. With our RTFE based approach for individualized load application considering differences in defect geometry and healing progression, the femur defect loading model could also be used to study the bone regeneration capacity of biomaterials under load application. With further advents in transcriptomics and registration techniques for different imaging technologies, the tightly controlled femur defect loading model could in the future widen our knowledge on the local spatio-temporal mechano-molecular regulation of the different fracture healing phases relevant for application of mechanical intervention therapies to clinical settings for improvement of impaired healing conditions.

## Methods

### Study design

By combining a novel in vivo femur defect loading model with a recently established time-lapsed in vivo micro-CT based monitoring approach, the influence of individualized cyclic mechanical loading on callus properties was assessed during the remodelling phase of fracture healing. All mice received a femur defect using a 0.66 mm Gigli wire saw and post-operative micro-CT scans (vivaCT 40, ScancoMedical, Brüttisellen, Switzerland) were performed. After bridging of the femur defect (week 3), RTFE allowed determination of initial loads (8 N–12 N), and weekly load-scaling and matching of strain distribution in the mineralized callus for the 4 week loading period. The loading group (n = 10) received individualized cyclic mechanical loading (8–16 N, 10 Hz, 5 min, 3 × /week) whereas controls (n = 10) received 0 N for 5 min at the same post-operative time-points. By registration of consecutive scans, structural and dynamic callus parameters were followed in three callus sub-volumes (defect centre: DC, defect periphery: DP, cortical fragment periphery: FP) and the adjacent cortical fragments (FC) over time (Fig. 3, for details on methods see Tourolle né Betts et al.^27^; for detailed study design see Supplementary Table S1).

### Animals

All animal procedures were approved by the Commission on Animal Experimentation (license number: 181/2015; Kantonales Veterinäramt Zürich, Zurich, Switzerland). We confirm that all methods were carried out in accordance with relevant guidelines and regulations (Swiss Animal Welfare Act and Ordinance (TSchG, TSchV)) and reported considering ARRIVE guidelines. Animal experiments were performed using previously established protocols for osteotomy surgery^25^, in vivo micro-CT imaging^26^, analgesia/anaesthesia^25,26^ and post-operative monitoring^25^. To study adult fracture healing female 12 week-old C57BL/6J mice were purchased from Janvier (Saint Berthevin Cedex, France) and housed in the animal facility of the ETH Phenomics Center (EPIC; 12 h:12 h light–dark cycle, maintenance feed (3437, KLIBA NAFAG, Kaiseraugst, Switzerland), 5 animals/cage) for 8 weeks. At an age of 20 weeks, all animals received a femur defect by performing an osteotomy with a 0.66 mm Gigli wire saw as previously described^26^ (group 1: control group, n = 10; group 2: loading group, n = 10; housing after surgery: 2–3 animals/cage; for details on study design see Supplementary Table 1). All defect surgeries were performed by the same veterinarian. Perioperative analgesia (25 mg/L, Tramal^®^, Gruenenthal GmbH, Aachen, Germany) was provided via the drinking water two days before surgery until the third post-operative day. For surgery and micro-CT scans, animals were anaesthetized with isoflurane (induction/maintenance: 5%/1–2% isoflurane/oxygen). Perioperative handling, and monitoring, micro-CT imaging and loading application was performed by the surgeon.

### Loading fixator assembly and group assignment

The four parts of the loading fixators (n = 20) were assembled as depicted in Fig. 1. To allow optimal identification throughout the experiments, one side part was engraved with a fixator-specific number (Fig. 1a,b). The stiffness of each fixator was measured using a Zwick testing machine and the fixators were assigned to the two groups, to allow similar distributions of fixator stiffness in the loading and control group (Supplementary Fig. S4).

### Femur osteotomy

In all animals an external fixator (Mouse ExFix, RISystem, Davos, Switzerland; mean stiffness: 17 N/mm; Supplementary Fig. 4) was positioned at the craniolateral aspect of the right femur and attached using four mounting pins. First, the most proximal pin was inserted approximately 2 mm proximal to the trochanter, followed by placement of the most distal and the inner pins. Subsequently, a femur defect was created using a Gigli wire (diameter: 0.66 mm).

### Time-lapsed in vivo micro-CT

Immediate post-surgery correct positioning of the fixator and the defect was visualized using a vivaCT 40 (Scanco Medical AG, Brüttisellen, Switzerland) (isotropic nominal resolution: 10.5 µm; 2 stacks of 211 slices; 55 kVp, 145 µA, 350 ms integration time, 500 projections per 180°, 21 mm field of view (FOV), scan duration ca. 15 min). Subsequently, the fracture callus and the adjacent bone between the inner pins of the fixator were scanned weekly using the same settings. Scans were registered consecutively using a branching scheme (registration of whole scan for bridged defects; separate registration of the two fragments for unbridged defects). Subsequently, morphometric indices (bone volume—BV, bone volume/total volume—BV/TV, bone formation rate – BFR, bone resorption rate—BRR) were computed (threshold: 395 mg HA/cm^3^; for details on methods see Tourolle né Betts et al.^27^). To assess mineralization progression, a second threshold (645 mg HA/cm^3^) was applied and the ratio between highly and lowly mineralized tissue (BV_645_/BV_395_) was calculated. The two selected thresholds are included in our recently developed multidensity threshold approach^27^. According to the standard clinical evaluation of X-rays, the number of bridged cortices per callus was evaluated in two perpendicular planes (UCT Evaluation V6.5-1, Scanco Medical AG, Brüttisellen, Switzerland). A ‘‘healed fracture’’ was considered as having a minimum of at least three bridged cortices per callus.

For evaluation, four volumes of interest (VOIs) were defined, which were created automatically from the post-operative measurement as described in Tourolle né Betts et al.^27^ (Fig. 3): defect centre (DC) containing endosteal callus and newly formed cortices, defect periphery (DP) containing the central periosteal callus, cortical fragment centre (FC) containing the medullary cavity and old cortices on both sides of the defect, and fragment periphery (FP) containing the periosteal callus adjacent to the old cortices. Data were normalised to the central VOIs: DC/DC, DP/DC, FC/FC, FP/FC.

Defect sizes (h) for each animal were calculated in MATLAB (version R2018b) based on the DC volume and the cross-sectional area of the proximal (CSAP) and distal cortices (CSAD): ${\text{h}} = \frac{2DC }{{\left( {CSAP + CSAD} \right)}}.$

### Individualized cyclic mechanical loading

From week 4 to week 7, individualized cyclic loading (8–16 N, 10 Hz, 3000 cycles; 3 × /week; controls—0 N) was applied via the external loading fixator (Fig. 1b) based on computed strain distribution in the callus using animal-specific RTFE analysis (for detailed description of methods see^24^). Briefly, after weekly micro-CT measurements of each animal, the images were pre-processed using threshold-binning to create a high-resolution multi-density FE mesh, which was then solved on a supercomputer within the same anaesthetic session for each mouse. 2D and 3D visualizations of the mineralized callus were generated and a strain histogram was plotted. This information allowed load scaling based on the strains induced in the mineralized callus in individualized animals. Specifically, the distribution was scaled to achieve a median effective microstrain of 700. To assess regions that posed a failure risk the 99th percentile strains of each simulation was visualised in both image based and histogram forms. If more than 50 voxels exceeded 1% then the optimised load was reduced by 1 N.

### Mechanical testing

3 point bending tests were performed on a Zwick compression tester (ZwickRoell GmbH & Co, Ulm, Germany). Tests were conducted on a setup of 8 mm support span and up to 3 N. Tests were performed quasi-statically with a cross-head speed of 0.4 mm/min. Stiffness was then calculated from the linear region of the resultant force–displacement curve. Each sample was tested three times and the mean stiffness was taken.

### Histology

Histological stainings were performed in one randomly selected animal per group. On day 49, femora were excised, the femoral head was removed and the samples were placed in 4% neutrally buffered formalin for 24 h and subsequently decalcified in 12.5% EDTA for 10–14 days. The samples were embedded in paraffin and the complete fracture callus was cut in 10 µm longitudinal serial sections. Every 10th section was stained with Safranin-O: Weigert’s iron haematoxylin solution (HT1079, Sigma-Aldrich, St. Louis, MO)—4 min, 1:10 HCl-acidified 70% ethanol—10 s, tap water—5 min, 0.02% Fast Green (F7258, Sigma-Aldrich, St. Louis, MO)—3 min, 1% acetic acid—10 s, 0.1% Safranin-O (84,120, Fluka, St. Louis, MO)—5 min. Images were taken with Slide Scanner Pannoramic 250 (3D Histech, Budapest, Hungary) at 20× magnification. Sections in between were stained for Sclerostin and RANKL.

Immunohistochemical staining of Sclerostin was performed according to Wehrle et al.^18^. Nonspecific sites were blocked (1% BSA/PBS + 1% rabbit serum) for 60 min at room temperature. Subsequently, the sections were incubated with the primary antibody against Sclerostin (AF1589, R&D Systems, Minneapolis, MN; 1:150 in 1%BSA/PBS + 0.2% rabbit serum) overnight at 4 °C. To detect the primary antibody, a secondary biotinylated rabbit anti-goat-IgG antibody (BAF017, R&D Systems, Minneapolis, MN) was added for 1 h at room temperature. For signal amplification, the slides were incubated with avidin–biotin complex (PK-6100 Vector Laboratories, Burlingame, CA) for 30 min. Diaminobenzidine (Metal Enhanced DAB Substrate Kit, 34,065 ThermoFisher Scientific, Waltham, MA) was used as detection substrate. Counterstaining was performed with FastGreen (F7258, Sigma-Aldrich, St. Louis, MO).

For immunohistochemical staining of RANKL, slides were placed into TBS-Tween buffer and inserted into the Dako-Autostainer (Dako, Ft. Collins, USA) using the following staining protocol: incubation with primary antibody against RANKL (1:100; ab 9957, Abcam, Cambridge, UK) at 4 °C overnight, rinsing with TBS-Tween, peroxidase blocking for 10 min at room temperature, rinsing with TBS-Tween, Envision + System HPR Rabbit (Dako K4003) for 60 min at room temperature, rinsing with TBS-Tween and incubation with DAB (Dako K3468) for 10 min at room temperature. The slides were rinsed in A. dest and counterstained with FastGreen for 2 s.

For both immunohistochemical stainings, species-specific IgG was used as isotype control (Supplemetary Fig. S5). Images were taken with Slide Scanner Pannoramic 250 (3D Histech, Budapest, Hungary) at 40 × magnification.

### Statistics

Data were tested for normal distribution (Shapiro–Wilk-Test) and homogeneity of variance (Levene-Test). Depending on the test outcome, group comparisons (loading vs. control group) of data derived at single time points (VOI size) were performed by two-tailed Student’s *t* test or Mann–Whitney U-test (IBM SPSS Statistics Version 23). For statistical evaluation of repeated measurements two-way ANOVA with Geisser-Greenhouse correction and Bonferroni correction (GraphPad Prism 8) were performed. The level of significance was set at *p* < 0.05.

## Supplementary Information

Supplementary Information.

## Data Availability

All necessary data generated or analysed during the present study are included in this published article and its Supplementary Information files (preprint available on BioRxiv (BIORXIV/2020.09.15.297861). Additional data that support the findings of this study are available from the corresponding author upon reasonable request.
