# Supplementary material for: Individualized cyclic mechanical loading improves callus properties during the remodelling phase of fracture healing in mice as assessed from time-lapsed in vivo imaging
Source: Sci Rep. 2021 Nov 29;11:23037. doi: 10.1038/s41598-021-02368-y (PMC8630002; doi:10.1038/s41598-021-02368-y)
Supplement: Supplementary file 1 — Supplementary Information. [file 41598_2021_2368_MOESM1_ESM.pdf]

Individualized cyclic mechanical loading improves callus properties during the remodelling phase of fracture healing in mice as assessed from time-lapsed *in vivo* imaging

Esther Wehrle[1*], Graeme R Paul[1], Duncan C Tourolle né Betts[1], Gisela A Kuhn[1], Ralph Müller[1*]

[1] Institute for Biomechanics, ETH Zurich, Zurich, Switzerland.

Corresponding authors:

Esther Wehrle, PhD, DVM

Institute for Biomechanics, ETH Zurich

Leopold-Ruzicka-Weg 4

8093 Zurich, Switzerland

Email: esther.wehrle@hest.ethz.ch

Ralph Müller, PhD

Institute for Biomechanics, ETH Zurich

Leopold-Ruzicka-Weg 4

8093 Zurich, Switzerland

Email: ram@ethz.ch

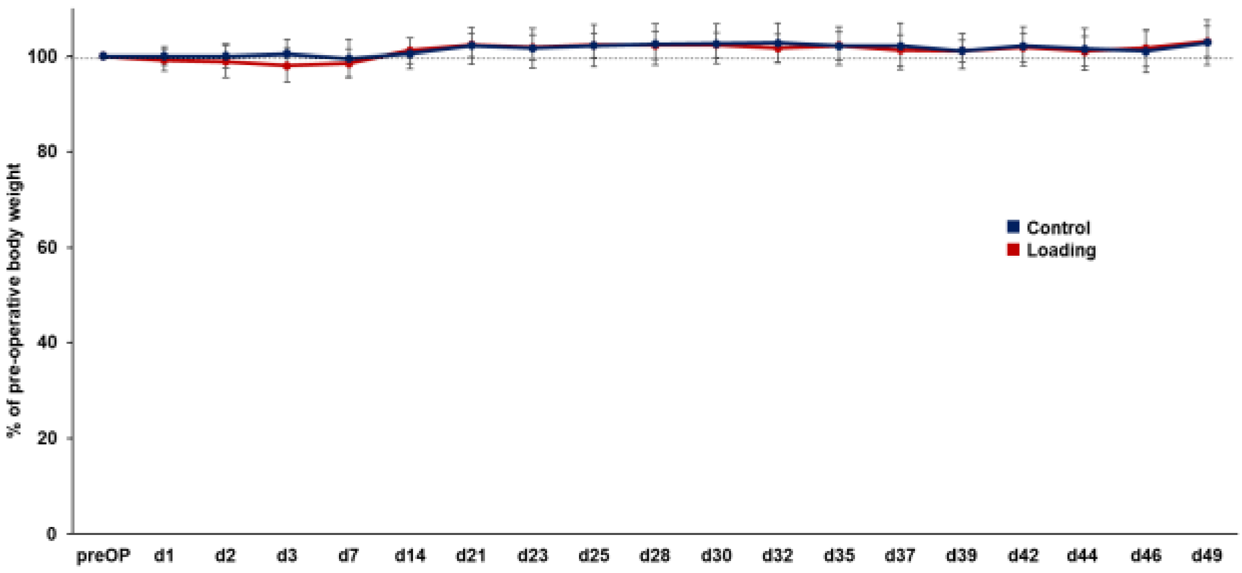

Supplementary Fig. S1. *In vivo* monitoring of body weight of the mice from the control group (n=10) and the loading group (n=10) measured pre-operatively (preOP), on postoperative days 1-3, weekly from day 7 to day 14 and 3x/week during the loading period (day 21 - day 49). The postoperative values were related to the preoperative data.

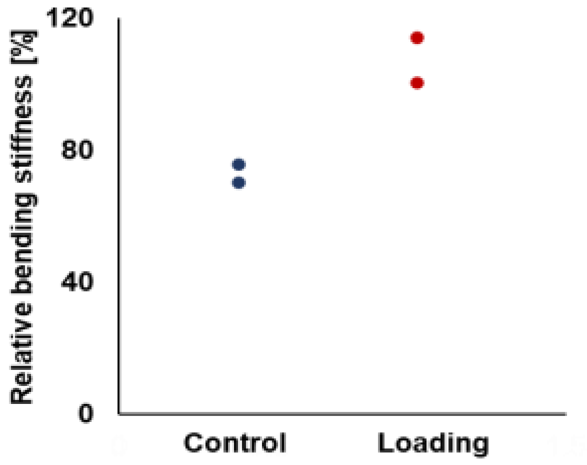

Supplementary Fig. S2. Relative bending stiffness (healed femur vs. contralateral femur) was assessed via *ex vivo* 3-point-bending testing for two animals from the loading and the control group.

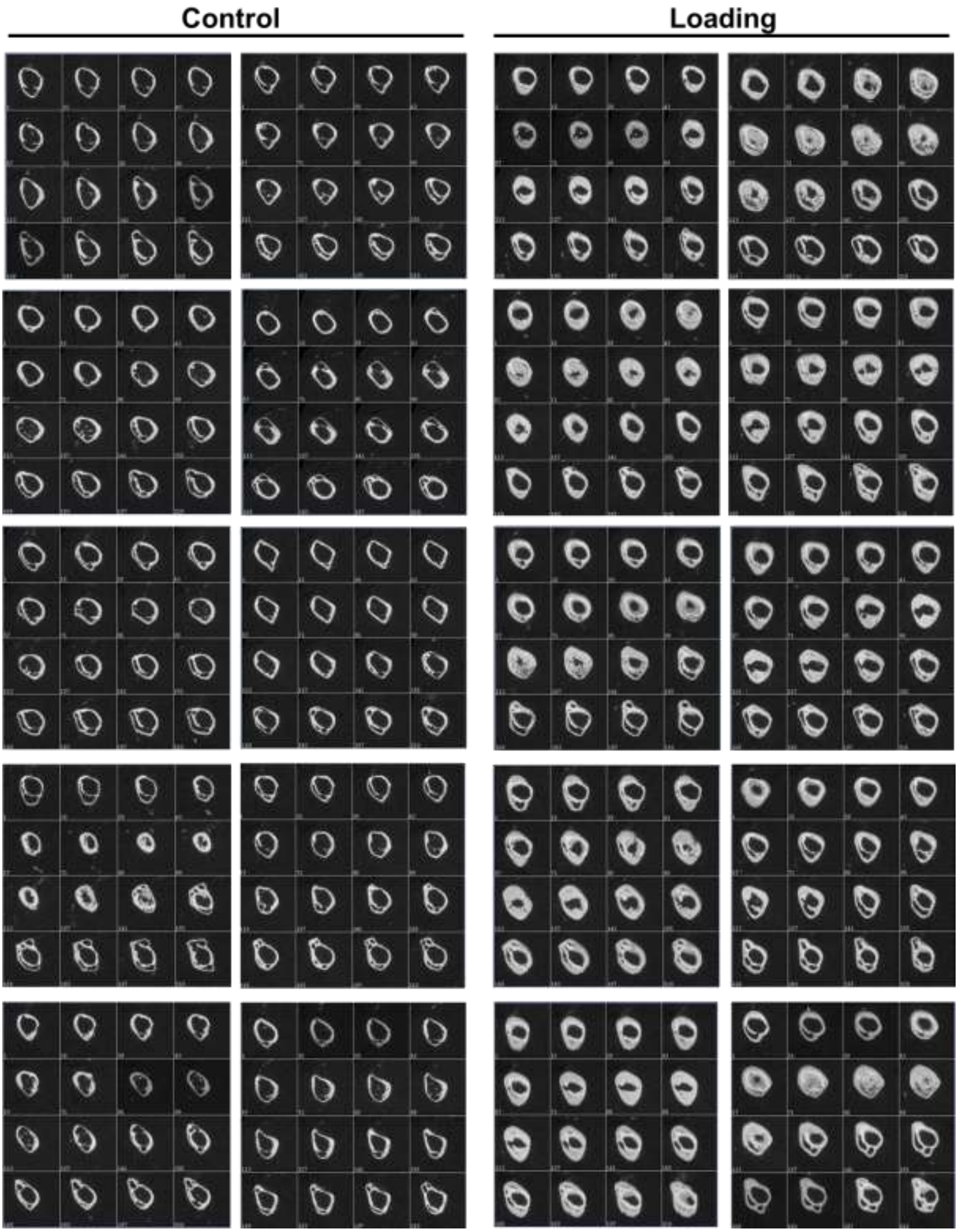

Supplementary Fig. S3. *In vivo* micro-CT scans of all 10 mice from the control and the loading group at day 49 post-osteotomy. For each animal 16 cross-sections of the mineralized tissue within the total VOI are shown. The distance between cross-sections is 147 µm.

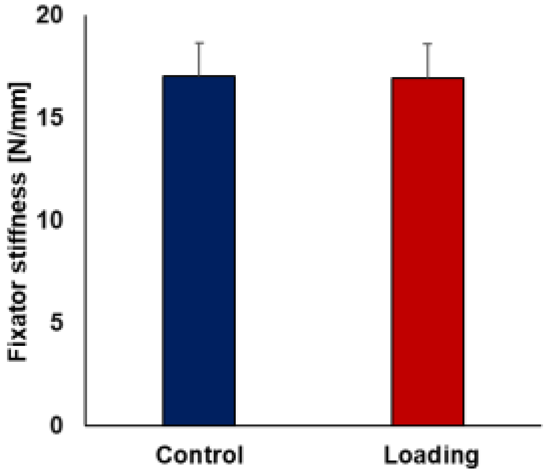

Supplementary Fig. S4. Pre-operative measurements of fixator stiffness were used to assign fixators to the loading group (n=10) and the control group (n=10).

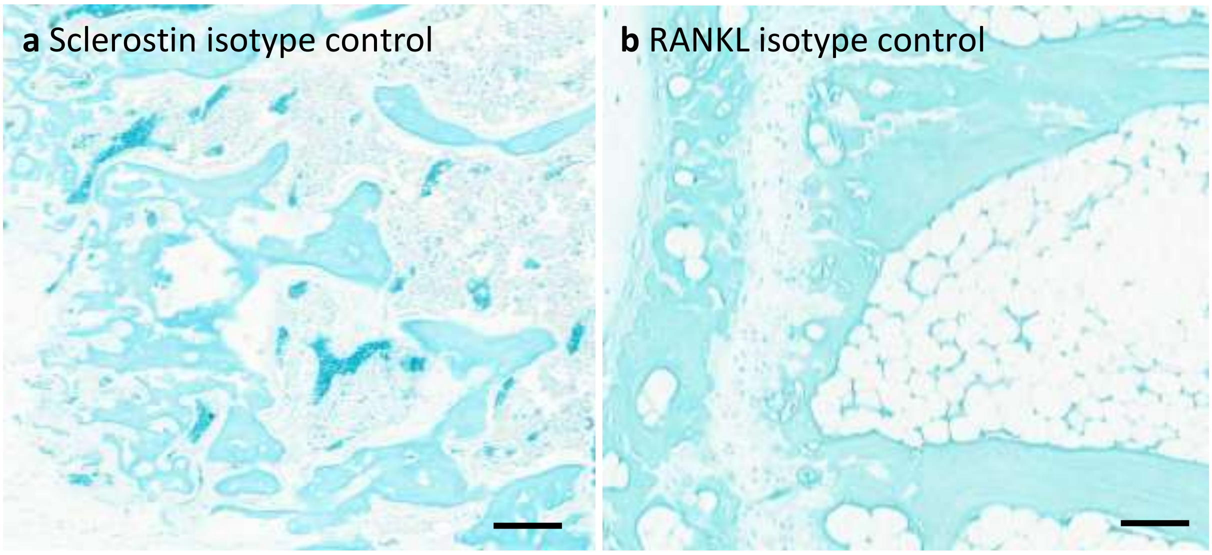

Supplementary Fig. S5. Images of isotype controls for Sclerostin and RANKL immunohistochemical stainings. a. Sclerostin isotype control, b. RANKL isotype control. Scale bar = 100 µm.

Supplementary Table S1. Study design (female 20 week-old C57BL/6J mice)

Group	Femur defect saw size: 0.66mm	*In vivo* micro-CT measurements	Registration of micro-CT scans #	Loading	Histology
Control	n=10	d0, week 1-7 (n=10)	week 1-7 (n=10)	0N, 3/week, week 3-7 (n=10)	week 7 (n=1)
Loading	n=10	d0, week 1-7 (n=10)	week 1-7 (n=10)	8-16N, 10 Hz, 3/week, week 3-7 (n=10)	week 7 (n=1)

micro-CT scan taken at timepoint x registered to micro-CT scan taken at timepoint x-1
